# Association of *Ugrp2* gene polymorphisms with adenoid hypertrophy in the pediatric population^☆^

**DOI:** 10.1016/j.bjorl.2017.07.004

**Published:** 2017-08-01

**Authors:** Mahmut Huntürk Atilla, Sibel Özdaş, Talih Özdaş, Sibel Baştimur, Sami Engin Muz, Işılay Öz, Kenan Kurt, Afife İzbirak, Mehmet Ali Babademez, Nilgün Vatandaş

**Affiliations:** a Yıldırım Beyazıt University, Yenimahalle Education and Research Hospital, Otolaryngology Clinic, Ankara, Turkey; b Adana Science and Technology University, Faculty of Engineering and Natural Sciences, Department of Bioengineering, Adana, Turkey; c Hacettepe University, Faculty of Science, Department of Biology, Ankara, Turkey; d Yıldırım Beyazıt University, Atatürk Education and Research Hospital, Otolaryngology Clinic, Ankara, Turkey; e Yıldırım Beyazıt University, Yenimahalle Education and Research Hospital, Pediatric Clinic, Ankara, Turkey

**Keywords:** Adenoid hypertrophy, Asthma, Allergy, *Ugrp2*, Single nucleotide polymorphism, Hipertrofia de adenoide, Asma, Alergia, *Ugrp2*, Polimorfismo de nucleotídeo único

## Abstract

**Introduction:**

Adenoid hypertrophy is a condition that presents itself as the chronic enlargement of adenoid tissues; it is frequently observed in the pediatric population. The *Ugrp2* gene, a member of the secretoglobin superfamily, encodes a low-molecular weight protein that functions in the differentiation of upper airway epithelial cells. However, little is known about the association of *Ugrp2* genetic variations with adenoid hypertrophy.

**Objective:**

The aim of this study is to investigate the association of single nucleotide polymorphisms in the *Ugrp2* gene with adenoid hypertrophy and its related phenotypes.

**Methods:**

A total of 219 children, comprising 114 patients suffering from adenoid hypertrophy and 105 healthy patients without adenoid hypertrophy, were enrolled in this study. Genotypes of the *Ugrp2* gene were determined by DNA sequencing.

**Results:**

We identified four single nucleotide polymorphisms (*IVS1-189G*>*A*, *IVS1-89T*>*G*, *c.201delC*, and *IVS2-15G*>*A*) in the *Ugrp2* gene. Our genotype analysis showed that the *Ugrp2* (*IVS1-89T*>*G*) TG and (*c.201delC*) CdelC genotypes and their minor alleles were associated with a considerable increase in the risk of adenoid hypertrophy compared with the controls (*p* = 0.012, *p* = 0.009, *p* = 0.013, and *p* = 0.037, respectively). Furthermore, *Ugrp2* (*GTdelCG*, *GTdelCA*) haplotypes were significantly associated with adenoid hypertrophy (four single nucleotide polymorphisms ordered from 5′ to 3′; *p* = 0.0001). Polymorfism–Polymorfism interaction analysis indicated a strong interaction between combined genotypes of the *Ugrp2* gene contributing to adenoid hypertrophy, as well as an increased chance of its diagnosis (*p* < 0.0001). In addition, diplotypes carrying the mutant *Ugrp2* (*c.201delC*) allele were strongly associated with an increased risk of adenoid hypertrophy with asthma and with allergies (*p* = 0.003 and *p* = 0.0007, respectively).

**Conclusion:**

Some single nucleotide polymorphisms and their combinations in the *Ugrp2* gene are associated with an increased risk of developing adenoid hypertrophy. Therefore, we tried to underline the importance of genetic factors associated with adenoid hypertrophy and its related clinical phenotypes.

## Resumo

**Introdução:**

A adenoide ou hipertrofia de tonsila faríngea é uma condição que se apresenta como o aumento crônico de tecidos linfóides na rinofaringe e é frequentemente observada na população pediátrica. O gene *Ugrp2*, um membro da superfamília da secretoglobina, codifica uma proteína de baixo peso molecular que funciona na diferenciação das células epiteliais das vias aéreas superiores. No entanto, pouco se sabe sobre a associação de variações genéticas do Ugrp2 com hipertrofia de tonsila faríngea.

**Objetivo:**

Investigar a associação de polimorfismos de nucleotídeos únicos no gene *Ugrp2* com hipertrofia de tonsila faríngea e seus fenótipos relacionados.

**Método:**

Foram incluídos no estudo 219 crianças, 114 pacientes com hipertrofia de tonsila faríngea e 105 saudáveis. Os genótipos do gene *Ugrp2* foram determinados por sequenciamento de DNA.

**Resultados:**

Identificamos quatro polimorfismos de nucleotídeo único (*IVS1-189G*>*A*, *IVS1-89T*>*G*, *c.201delC*, e *IVS2-15G*>*A*) no gene *Ugrp2*. Nossa análise genotípica mostrou que os genótipos *Ugrp2* (*IVS1-89T*>*G*) TG e (*c.201delC*) CdelC e seus alelos menores foram associados a um aumento considerável no risco de HA em comparação com os controles (*p* = 0,012, *p* = 0,009, *p* = 0,013 e *p* = 0,037, respectivamente). Além disso, os haplótipos *Ugrp2* (*GTdelCG, GTdelCA*) foram significativamente associados com hipertrofia de tonsila faríngea (quatro polimorfismos de nucleot’ ordenados de 5’ a 3’; *p* = 0,0001). A análise de interação polimorfismo-polimorfismo indicou uma forte interação entre genótipos combinados do gene *Ugrp2* que contribuiu para hipertrofia de tonsila faríngea, bem como uma chance maior de seu diagnóstico (*p* < 0,0001). Além disso, os diplótipos que transportam o alelo mutante *Ugrp2* (*c.201delC*) foram fortemente associados a um risco aumentado de hipertrofia de tonsila faríngea com asma e com alergias (*p* = 0,003 e *p* = 0,0007, respectivamente).

**Conclusão:**

Alguns polimorfismos de nucleotídeo único e suas combinações no gene *Ugrp2* estão associados a um risco aumentado de desenvolver hipertrofia de tonsila faríngea. Portanto, tentamos enfatizar a importância dos fatores genéticos e fenótipos clínicos associados à essa hipertrofia.

## Introduction

The adenoids, also known as the pharyngeal or nasopharyngeal tonsils, are important components of the Waldeyer's ring. They appear to play an important role in the development of “immunological memory”.^1^ Adenoid hypertrophy (AH) is a very common condition in children.^2^ Symptoms associated with AH include nasal congestion, rhinorrhea, hyponasal speech, open-mouthed breathing, witnessed sleep apnea, snoring, and a facial structure known as “adenoid face” caused by chronic airway obstruction.^2^, ^3^ The exact mechanisms underlying follicular lymphoid proliferation and hyperplasia of the adenoids are poorly understood. However, it is currently thought that recurrent or chronic inflammation of the adenoids contributes to the chronic activation of cell-mediated and humoral immune responses, leading to AH.^4^

Cytokines, which function in immunological responses, may affect the process of inflammation. In addition, certain single nucleotide polymorphisms (SNPs) in cytokines are thought to contribute to a predisposition to AH.^5^ Defining the factors that affect AH-associated immune responses would provide important data to determine the pathogenesis of AH. Various genetic factors may affect certain disease phenotypes and change the severity of chronic inflammatory diseases by altering gene expression levels.^6^

Secretoglobins (SCGBs) are a newly discovered and rapidly growing physiologically and pathophysiologically active superfamily of cytokine-like secretory proteins with small dimeric structures. They are candidates for a new cytokine family, as they have anti-inflammatory and immunomodulatory functions. Eleven SCGB genes and five pseudogenes have been described in the human genome.^7^ The *Ugrp2* (*Scgb3a1*) gene was reported to play a role in the pathogenesis of some cancers, as well as upper and lower airway inflammatory diseases including asthma, allergies, and nasal polyposis.^8^, ^9^, ^10^ It was also reported that UGRP2 is a potent inhibitor of cell growth, migration, and invasion via the AKT signaling pathway.^9^

The *Ugrp2* gene was identified in 2002; however, very few studies have been performed on this gene.^7^ Therefore, in the current study, we aimed to identify SNPs in the *Ugrp2* gene and to analyze the associations of those SNPs with AH in children.

## Methods

### The study population

The study cohort in this investigation consisted of 114 children who were admitted to the Ear-Nose-Throat (ENT) and pediatric clinics with symptoms of open-mouthed sleeping, snoring, witnessed sleep apnea, hyponasal speech, malnutrition, and midfacial development disorders and were diagnosed with AH. The diagnosis of AH was based on patient history as well as physical and endoscopic examinations.^11^ AH was graded in accordance with Parikh's classification: Grade 1 indicates inflammation of an adenoid tissue not in contact with any adjacent structures; Grade 2 indicates inflammation of an adenoid tissue in contact with the torus tubarius; Grade 3 indicates inflammation of an adenoid tissue in contact with the vomer; and Grade 4 indicates inflammation of an adenoid tissue in contact with the soft palate.^12^ In our study, patients classified with Parikh's Grades 2, 3, or 4 were regarded to have AH, and were included in the study.

The control group consisted of 105 healthy children who were admitted to the outpatient clinic of the same hospital for regular follow-ups or minor traumas. Nasal endoscopic examinations were performed to confirm that they did not suffer from AH.

Confirmation of witnessed sleep apnea, snoring, and open-mouthed sleeping was obtained from the parents of the children.^13^ Asthma diagnosis was based on patient history or examination of the children in the pulmonology department.^14^ Skin-prick tests were used to identify allergies. The skin prick tests were performed in accordance with the recommendations of the European Academy of Allergology and Clinical Immunology (EAACI).^15^ Children whose results were positive for at least one of the allergens tested were considered allergic.

The parents of the children were informed about the study, and informed consent was obtained both verbally and in writing. The local ethics committee approved the study protocols, and the study was conducted in accordance with human rights and experimental ethics (registration number 2012-898).

Children with craniofacial abnormalities, congenital defects, mental retardation, or immunodeficiency, as well as cardiovascular, pulmonary, metabolic, genetic, or neuromuscular diseases were excluded from this study.^16^

### DNA sequencing

Blood samples were incubated in EDTA. Genomic DNA was extracted from peripheral blood leukocytes using the NucleoSpin DNA kit (Macherey-Nagel, Germany). PCR was performed to amplify the relevant gene region (SuperHot Master Mix, Bioron, Germany). The *Ugrp2* gene has three exons; therefore, three primer pairs were used for the PCR reaction. For the first exon, 5′-GGTCAGACCGCAAAGCGAAGG-3′ was used as the forward primer and 5′-GACCTGGGATCCACGATCGG-3′ was used as the reverse primer; a 465-bp fragment was amplified. For the second exon, 5′-TGCACAGAGTTCACCGGTCCTTC-3′ was used as the forward primer and 5′-AGGGGCAGGACGGGAAACAG-3′ was used as the reverse primer; a 611-bp fragment was amplified. For the third exon, 5′-CCGCTCCCGCTCCCCACAGA-3′ was used as the forward primer and 5′-TCTCTCCCTCTCTCACGCAGCAC-3′ was used as the reverse primer; a 349-bp fragment was amplified. The NucleoFast 96 PCR kit was used for purification of the amplified products (Macherey-Nagel). Sequencing was performed for *Ugrp2*. The sequencing reaction was performed using purified PCR products. The obtained sequence products were purified using the ZR Sequencing Clean-up Kit D4051 (Zymo Research, USA) and prepared for array analysis. Polymorphisms in *Ugrp2* were identified using the ABI PRISM 3130 Genetic Analyzer capillary automatic sequencing equipment.

### Statistical analysis

The Statistical Package for Social Sciences (SPSS) program (version 22.0, SSPS Inc., USA) was used for statistical analysis. The average age between groups was compared using the Mann–Whitney *U* test. The *χ*^2^ test or Fisher's exact test was used to analyze the association between SNPs of *Ugrp2* and clinical phenotypes. The frequency of each genotype was tested using *χ*^2^ for concordance with Hardy–Weinberg Equilibrium (HWE).^17^

A study by Aydin et al. on 1132 subjects in Turkey obtained a frequency of AH of 27% for 5–7 year-old children, and 19.5% for 8–10 year-old children.^18^ Taking the prevalence of AH into consideration, the smallest sample size required to achieve 78% confidence was determined to be 90 cases.^19^ Therefore, we enrolled 114 patients with AH and 105 healthy children to our study.

SNPStats was used to determine the degree of pairwise Linkage Disequilibrium (LD), as well as genotype and haplotype analysis (http://bioinfo.iconcologia.net/index.php?module=Snpstats).^20^ This software provides the odds ratio (OR) and 95% confidence interval (95% CI). We used the Multifactor Dimensionality Reduction (MDR) software package (version 1.0.0, available at www.epistasis.org) to address possible SNP–SNP and SNP–phenotype interactions. MDR is a novel approach described by Hahn et al.^21^ It is a computational tool and a non-parametric method (i.e. no parameters are estimated) that is able to detect SNP–SNP and SNP–phenotype interactions in genetic association studies. This method reduces data dimensionality by pooling genotypes from multiple SNPs into either high-risk or low-risk groups for a disease, thereby circumventing the problem of analyzing high-order genotype combinations with low numbers of observations. The software successfully identified disease-associated multi-locus genotype combinations and discrete environmental factors.^22^, ^23^ *p* < 0.05 was considered significant in all statistical analyses.

## Results

### Characteristics of the study participants

A total of 219 children (114 patients with AH comprising 54 females and 60 males with a mean age of 5.38 years [3–9 years)] and 105 healthy children comprising 46 females and 59 males with a mean age of 5.23 years [2–10 years]) were included in this study. The mean ages (*p* = 0.570) and gender distributions (*p* = 0.709) between the study and control groups were not statistically significant. Other relevant characteristics of the study population are summarized in Table 1.

**Table 1:** Characteristics of the study population.

	Patients with AH (*n* = 114)	Healthy control children (*n* = 105)	*p*-Value
*Age (years) (mean* *±* *SD)*	5.38 (1.532)	5.23 (1.712)	0.570^a^
*Gender, n (%)*			
Male	60 (53)	59 (56)	0.709^b^
Female	54 (47)	46 (44)	

*Asthma (+), n (%)*	30 (26)	14 (13)	**0.042**^b^
*Allergy (+), n (%)*	33 (29)	15 (14)	**0.032**^c^

Values are presented as median ± SD or numbers (%) unless otherwise specified.

AH, adenoid hypertrophy; SD, standard deviation; *n* (%), frequency.

The bold values mean statistically significant.

a Mann–Whitney *U* test.

b Fisher exact test.

c *χ*^2^ test.

The prevalence of asthma and allergies was higher in children with AH than in the control group (*p* = 0.047 and *p* = 0.032, respectively). In the AH group, 76 (67%) children had a history of open-mouthed sleeping, 80 children (70%) had a history of snoring, and 81 children (71%) had a history of witnessed sleep apnea.

### Relationship between Ugrp2 and AH

In the current study, we detected four SNPs within the *Ugrp2* gene. The SNPs *IVS1-189G*>*A*, *IVS1-89T*>*G*, *c.201delC*, and *IVS2-15G*>*A* were observed within intron 1, intron 1, exon 2, and intron 2 of the gene, respectively. The SNPs were reported previously, and have been registered in the SNP database (dbSNP; Short Genetic Variations Database at http://www.ncbi.nlm.nih.gov/snp; accession numbers rs549066053, rs117170752, rs200868132, and rs529004193, respectively). The genotypic distributions of the healthy children were consistent with those expected according to HWE calculations (*p* > 0.05 for all).

As shown in Table 2, genotype analyses revealed that the frequencies of the *Ugrp2* (*IVS1-189G*>*A*) GG, GA genotype and minor allele were similar between the study and control groups (0.02 vs. 0.02; *p* = 0.092 and *p* = 1, respectively). This SNP showed no correlation with an increased risk of AH. In addition, we found that the frequencies of the *Ugrp2* (*IVS1-89T*>*G*) TT, TG genotype and G allele were different between the study and control groups (0.07 vs. 0.03; *p* = 0.012 and *p* = 0.013, respectively). Moreover, individuals with the *Ugrp2* (*IVS1-89T*>*G*) TG genotype and G allele were 2.5- or 2.3-times more likely to suffer from AH than those with the TT genotype and T allele. Furthermore, we found that frequencies of the *Ugrp2* (*c.201delC*) CC, CdelC genotype, and delC allele were significantly different between the study and control groups (0.003 vs. 0; *p* = 0.0009 and *p* = 0.037, respectively). Individuals with the *Ugrp2* (*c.201delC*) CC, CdelC genotype, and delC allele were also 5- and 4.6-times more likely to suffer from AH than those with the homozygous wild-type genotype and major allele. The frequencies of the *Ugrp2* (*IVS2-15G*>*A*) GG, GA genotype and minor allele were not significantly different between the study and the control groups (0.001 vs. 0; *p* = 0.25, *p* = 1, respectively).

**Table 2:** Frequencies of *UGRP2* SNPs genotypes and alleles.

SNPs	Genotype/allele	Controls (*n* = 105)	Cases (*n* = 114)	OR (95% CI)	*p*-Value
		*n* (%)	*n* (%)		
*IVS1-189G*>*A*	G/G	101 (96)	109 (96)	1.00 (reference)	0.092^a^
	G/A	4 (4)	5 (4)	1.00 (0–0)	
	A/A	0 (0)	0 (0)	–	
	A^c^	0.02	0.02	1.00 (0–0)	1^b^
*IVS1-89T*>*G*	T/T	99 (94)	99 (87)	1.00 (reference)	**0.012**^a^
	T/G	6 (6)	15 (13)	**2.50 (0.75**–**8.37)**	
	G/G	0 (0)	0 (0)	–	
	G^c^	0.03	0.07	**2.39 (0.79–7.81)**	**0.013**^a^
*201delC*	C/C	105 (100)	106 (93)	1.00 (reference)	**0.0097**^a^
	C/delC	0 (0)	8 (7)	**5 (1.25**–**20)**	
	delC/delC	0 (0)	0 (0)	–	
	delC^c^	0.00	0.003	**4.68 (1.0**–**9.2)**	**0.037**^b^
*IVS2-15G*>*A*	G/G	105 (100)	113 (99)	1.00 (reference)	0.25^a^
	G/A	0 (0)	1 (1)	0.99 (0.07–10)	
	A/A	0 (0)	0 (0)	–	
	A^c^	0.00	0.01	0.99 (0.8–11.15)	1^a^

*n* (%), frequency; SNP, single nucleotide polymorphism; OR, odds ratio; CI, confidence interval; Del, deletion.

The bold values mean statistically significant.

a *χ*^2^ test.

b Fisher exact test.

c Assumed risk alleles.

In our study, we found that the *Ugrp2* (*c.201delC*) and (*IVS2-15G*>*A*) SNPs showed the highest pairwise LD (*r*′ = 0.434511). Haplotype analysis of all four SNPs was performed, and it was found that some haplotypes showed a strong correlation with AH (*p* = 0.049) (Table 3). We found that the GTdelCG and GTdelCA haplotypes were a potential risk factor for patients with AH (*p* = 0.0001 and *p* = 0.0001, respectively), while the GTCG haplotype was found in both groups.

**Table 3:** Associations of AH risk and frequencies of haplotypes on the basis of the SNPs observed in *UGRP2* in the AH group and controls.

N°	Haplotypes^a^				Frequencies		OR (95% CI)	*p*-Value
	*IVS1-189G*>*A*	*IVS1-89T*>*G*	*c.201delC*	*IVS2-15G*>*A*	Cases	Controls		
1	G	T	C	G	0.8816	0.9500	1.00	–
2	G	G	C	G	0.0658	0.0286	2.72 (0.81–9.14)	0.011
3	A	T	C	G	0.0197	0.0214	1.09 (0.21–5.60)	0.92
4	G	T	DelC	G	0.0263	0	2528.32 (2523.00–2530.64)	<0.0001
5	G	T	DelC	A	0.0066	0	2523.96 (2523.88–2523.05)	<0.0001

OR, odds ratio; CI, confidence interval; AH, adenoid hypertrophy.

a The alleles of the haplotypes were arrayed as the location of in the SNPs *UGRP2*. Global haplotype association *p*-value = 0.049.

### The relationship between Ugrp2 and AH-related phenotypes

Genotype analysis showed a strong correlation with AH phenotypes for some genotypes. The *Ugrp2* (*c.201delC*) CdelC and *Ugrp2* (*IVS1-89T*>*G*) TG SNPs were more commonly found in children with allergic-AH and asthmatic-AH than in the controls (OR = 2.1, 95% CI = 1.33–7.24, *p* = 0.006 and OR = 2.85, 95% CI = 1.34–6.10, *p* = 0.005, respectively). In addition, the *Ugrp2* (*IVS1-189G*>*A*) GG and (*IVS1-89T*>*G*) TG genotypes were more commonly found in AH patients with open-mouthed sleeping than in healthy children (OR = 7.08, 95% CI = 3.33–15.04, *p* = 0.28 and OR = 15.91, 95% CI = 1.84–137.19, *p* = 0.94, respectively). Moreover, the frequencies of the *Ugrp2* (*IVS1-189G*>*A*) GG and (*IVS1-89T*>*G*) TT, TG genotypes were more common in AH patients with witnessed sleep apnea than in the controls (OR = 7.54, 95% CI = 3.53–16.07, *p* = 0.56; OR = 9.01, 95% CI = 4.10–19.81, *p* = 0.31; and OR = 11.76, 95% CI = 1.23–112.67, *p* = 0.30, respectively). Logistic regression modeling showed that the frequencies of the *Ugrp2* (*IVS1-189G*>*A*) GG and (*IVS1-89T*>*G*) TG genotypes were higher in patients with snoring than in the control group (OR = 6.74, 95% CI = 3.21–14.14, *p* = 0.043 and OR = 16.95, 95% CI = 1.95–147.31, *p* = 0.88, respectively).

Haplotype analysis showed a significant relationship between the GGCG haplotype and asthma (OR = 7.16, 95% CI = 2.23–22.98, *p* = 0.0012). In addition, the GTdelCG and GTdelCA haplotypes were found in significantly higher frequencies in AH patients with snoring and witnessed sleep apnea than in control patients (for snoring: OR = 2787.87, 95% CI = 2786.45–2789.29, *p* = 0.0001 and OR = 2887.47, 95% CI = 27,881.41–2790.32, *p* = 0.000, respectively. For witnessed sleep apnea: OR = 2608.55, 95% CI = 2600.16–2670.05, *p* = 0.0001 and OR = 2618.84, 95% CI = 2601.16–26.45, *p* = 0.0001, respectively).

### SNP–SNP interactions

MDR analysis of the SNPs in the *Ugrp2* gene revealed statistically significant interactions (Table 4). Each optimized model across all possible combinations was assessed by Testing the Balanced Accuracy (TBA), Cross-Validation Consistency (CVC), and significance level. We found three predictive models for AH. The single-locus model for *Ugrp2* (*IVS1-89T*>*G*) was identified to be a reliable risk factor for prediction of AH. This single-locus model demonstrated the effects of the *Ugrp2* (*IVS1-89T*>*G*) TG polymorphism; the presence of this mutation increased the risk of AH 2.5-fold, and its minor allele was placed in the high-risk group for AH (OR = 2.50, 95% CI = 0.7465–8.3729). The two-locus model for *Ugrp2* (*IVS1-89T*>*G_c.201delC*) showed a slightly less convincing association with the diagnosis of AH. Homozygous wild-type genotypes as well as these SNPs were placed in the low-risk group, while the TG + CC and TT + CdelC diplotypes were placed in the high-risk group for AH (OR = 4.0574, 95% CI = 1.2763–12.8986). The three-locus model for *Ugrp2* (*IVS1-189G*>*A_IVS1-89T*>*G*_*c.201delC*) was identified as the third-best predictor of AH risk. Therefore, homozygous wild-type genotypes together with these SNPs were also placed in the low-risk group. However, the GG + TT + CdelC and GG + TG + CC triplotypes were placed in the high-risk group for AH (OR = 3.9375, 95% CI = 1.1936–12.9881).

**Table 4:** Multifactor dimensionality reduction analysis of the SNPs in *UGRP2.*

Model	TBT	TBA	CVC
*IVS1-89T*>*G*	0.5394	0.5043	7/9
*IVS1-89T*>*G_c.201delC*	0.9877	0.4862	9/10
*IVS1-189G*>*A*_*IVS1-89T*>*G*_*c.201delC*	0.9877	0.4862	10/10

TBT, Testing Balanced Training; TBA, Testing Balanced Accuracy; CVC, Cross-Validation Consistency.

*p*-Value < 0.0001.

Another interaction model, determined empirically by permutation testing, was the two-factor model analyzing the combination of the *Ugrp2* (*IVS1-189G*>*A*) and (*c.201delC*) alleles with asthma. These combinations were found to be associated with a high and low risk for AH with a TBA of 60.6% and a CVC of 10/10 (*p* = 0.003). However, GG + CdelC + asthma was placed in the high-risk group for AH (OR = 3.3973, 95% CI = 1.4172–8.1440). Furthermore, the combinations of the *Ugrp2* (*IVS1-89T*>*G*) and (*c.201delC*) alleles with allergies were found to be associated with a high and low risk for AH with a TBA of 63.2% and a CVC of 9/10 (*p* = 0.0007). However, TT + CdelC + allergy was placed in the high-risk group for AH (OR = 2.4681, 95% CI = 0.2329–26.1529).

## Discussion

In this study, we analyzed the associations between *Ugrp2* polymorphisms and certain phenotypes by screening a pediatric population with AH and without AH. UGRP2 (also called High In Normal-1 [HIN-1]) is a novel cytokine that induces the differentiation of epithelial cells and functions in negative cell growth.^9^, ^24^ To the best of our knowledge, our study is the first to evaluate the relationships between *Ugrp2* SNPs and AH risk in the Turkish pediatric population. The results of our study indicated significant association between certain genotypes, haplotypes, diplotypes, and triplotypes of the *Ugrp2* gene and the pathogenesis of AH, allergies, and asthma.

The *Ugrp2* gene is located on chromosome 5q35 of the human genome. Chromosome 5q31–36 contains an asthma susceptibility locus consisting of several genes associated with inflammation, certain cytokines, and the Granulocyte Macrophage Colony-Stimulating Factor-2 (GM-CSF2).^8^, ^25^ Expression of the *Ugrp2* gene is downregulated in nasal polyposis through the differentiation of human upper airway epithelial cells, suggesting that this gene has a role in the differentiation of mucinous epithelial cells such as adenoid tissues.^8^, ^26^

The UGRP1 protein is a member of the SCGB superfamily. UGRP1 and UGRP2 have certain similarities between their amino sequences, especially within the antiflammin domain, which is responsible for the anti-inflammatory and immunomodulatory activities of UGRP2, which possesses anti-inflammatory functions.^27^, ^28^ Recent studies have demonstrated that UGRP1 is strongly associated with asthma and allergic airway inflammation (http://omim.org/entry/606531).^29^, ^30^ Therefore, considering the current literature, we suggest that UGRP2 may also be associated with the pathogenesis of AH and its related phenotypes, such as asthma and allergies.

In the current study, we identified four SNPs in *Ugrp2* (*IVS1-189G*>*A*, *IVS1-89T*>*G*, *c.201delC*, and *IVS2-15G*>*A*). We also observed that children with AH carried a heterozygous mutation in *Ugrp2* (*IVS1-189G*>*A*). However, the frequency of this allele was low (4%) and was similar to the frequency observed in the healthy control group. Furthermore, we observed that the frequency of AH in patients bearing the *Ugrp2* (*IVS2-15G*>*A*) GA genotype was lower than in patients with the GG genotype; however, this difference was not statistically significant. The results of this study also indicated a 2.3-fold increase in AH risk in the presence of the G allele for *Ugrp2* (*IVS1-89T*>*G*) genotype compared to the T allele. The TG genotype was significantly more common in children with AH than in the controls, and appeared to be associated with a 2.5-fold increase in the risk of developing AH compared to the wild-type TT genotype. We also determined that the *Ugrp2* (*c.201delC*) mutation was associated with a 4.6-fold increase in the risk of developing AH. In addition, there were more children in the AH group with the *Ugrp2* (*c.201delC*) CdelC genotype than in the control group; those children were five times more likely to develop AH than children with the CC genotype.

AH can cause various comorbid conditions such as sleep apnea, atopic diseases, chronic serous otitis, and sinusitis.^2^, ^3^ Asthma and allergies are the most common atopic diseases in children with AH as they share a common genetic background, as well as similar immunological pathway.^3^, ^31^ Unsurprisingly, the frequency of allergies and asthma was higher in children with AH than in children without AH in this study. However, the presence of asthma and allergies in the control group may reflect multifactorial genetic and environmental etiologies for those disorders.^32^, ^33^ We also found that the *Ugrp2* (*c.201delC*) CdelC and (*IVS1-89T*>*G*) TG genotypes were the most common in children with allergic-AH and asthmatic-AH. In addition, a combination of *Ugrp2* (*IVS1-189G*>*A_c.201delC*) GG + CdelC + asthma and (*IVS1-89T*>*G_c.201delC*) TT + CdelC + allergies were associated with a 3.3- and 2.4-fold increase in AH risk, respectively. UGRP2 is a small secretory protein that is highly expressed in airway epithelial tissues.^8^, ^9^, ^10^ The expression of UGRP2 is associated with cytokines and proinflammatory cytokines that play a critical role in AH.^5^, ^6^, ^26^ Our results suggest that some SNPs and combinations of SNPs may affect the expression of *Ugrp2*, inducing changes in cytokine expression leading to a local immune response and upper airway inflammation; this increases the risk of asthma and allergic reactions in children with AH. Our findings are consistent with those reported previously, and illustrated the effects of *Ugrp2* on inflammation.^29^, ^30^ However, further in vitro functional analyses should be conducted on these children to investigate the development of AH in the future.

We have determined that the *Ugrp2* (*IVS1-189G*>*A*)/wild-type and (*IVS1-89T*>*G*)/wild-type heterozygous genotypes were the most common in patients with open-mouthed sleeping, sleep apnea, and snoring. On the other hand, a previous study found that the UGRP2 (IVS1-89) mutation was present in patients with NP and the control group at similar frequencies.^10^ It is possible that the SNPs in question, along with some environmental factors, may contribute to an inherited predisposition for AH and some of its phenotypes.

The sequencing results indicated that the *Ugrp2* (*c.201delC*) mutation is located on an exon, while the other three SNPs are located on introns. SNPs in non-coding regions affect pre-mRNA splicing; SNPs in the coding areas affect the amino acid sequence and play significant roles in human genetic diseases.^34^ In this context, the *Ugrp2* (*c.201delC*) (p.Val68fs) mutation is a frameshift mutation resulting from the deletion of one of three cytosine bases (CCC) usually present at position 226 of the mRNA sequence, leading to a frameshift after codon 68 [Valine (Val)] of the *Ugrp2* gene. The *Ugrp2* (*c.201delC*) mutation alters the translational reading frame of the putatively translated mRNA, as well as *Ugrp2*′ expression; the resulting mutant protein presumably changes the protein activity of UGRP2 (Q96QR1).^35^ Those results suggest that the *Ugrp2* (*IVS1-89T*>*G*) and (*c.201delC*) mutations may play a role in the pathophysiology of AH, allergies, and asthma. However, further studies are required in order to elucidate the importance of those SNPs in AH and its related phenotypes.

Haplotype-, diplotype-, and triplotype-based association analyses were more effective and informative than allele- and genotype-based analyses.^36^ In our study, haplotype association analysis revealed that the GTdelCG or GTdelCA haplotypes carrying the *Ugrp2* (*c.201delC*) delC allele were associated with a significantly higher risk for AH, snoring, and witnessed sleep apnea. This was possibly due to the fact that the *Ugrp2* (*c.201delC*) and (*IVS2-15G*>*A*) mutations were strongly linked with each other (*r*^2^ ≥ 0.33), indicating that this region might be inherited together.^37^

MDR is a model-free method (i.e. a method without any assumed genetic model) designed to detect SNP–SNP or SNP–phenotype interactions in case–control studies in the absence of significant effects. MDR has become one of the most effective programs for genetic association studies, and for understanding complex traits.^21^, ^22^, ^23^ For SNP–SNP interactions and their effects, genotype-, diplotype-, or triplotype-based association analyses are likely to be more informative than association analyses based on haplotypes, genotypes, or alleles. In the present study, MDR results suggested that the *Ugrp2* (*IVS1-89T*>*G*) TG genotype, the (*IVS1-89T*>*G_c.201delC*) TG + CC, TT + CdelC diplotypes, and the (*IVS1-189G*>*A_IVS1-89T*>*G_c.201delC*) GG + TT + CdelC, GG + TG + CC triplotypes were associated with a 2.5-, 2.5-, ∞-, ∞-, and 2.5-fold increased risk of AH in children, respectively. The previously mentioned results support a model showing that strong interactions between these polymorphisms and some phenotypes may lead to an increased susceptibility to AH.

Frequencies of *Ugrp2*-related SNPs in different healthy populations worldwide are available on online databases (www.hapmap.org and www.ncbi.nlm.nih.gov/snp), but their applicability is limited. However, the allele frequencies and rates of polymorphisms for this gene in healthy controls in our study were different from the frequencies reported in these databases, but were similar to a previous study performed in a Turkish population.^10^ These inconsistencies in the minor allele frequencies of *Ugrp2* polymorphisms among healthy individuals demonstrate the importance of geographic distribution, ethnicity, and sample size in association studies.^36^ The children included in this study lived in a similar geographical location. However, a potential limitation of our study is that we required a hospital-based cohort, and therefore lacked data on ethnic disparities and population stratification.

Another limitation of our study was the relatively small sample size. This is particularly important because phenotypic classifications with several selection criteria were applied for the selection of controls in this study. An additional limitation may be the inclusion of subjects clinically diagnosed with Parikh's Grade 2, 3, or 4 AH only, leading to the absence of any genetic analyses on patients with smaller adenoids. Considering the above limitations, further functional studies are required using a larger study group consisting of independent samples and family-based cohorts to elucidate the relationship between *Ugrp2* gene polymorphisms and AH.

## Conclusion

This study identified several SNPs in the *Ugrp2* gene that were associated with increased susceptibility for AH and AH-related clinical phenotypes. Our results highlighted the potential role of *Ugrp2* in the pathogenesis of AH, and serve as preliminary data for future studies.

## Conflicts of interest

The authors declare no conflicts of interest.
